# A CRR2-Dependent sRNA Sequence Supports Papillomavirus Vaccine Expression in Tobacco Chloroplasts

**DOI:** 10.3390/metabo13030315

**Published:** 2023-02-21

**Authors:** Julia Legen, Sara Dühnen, Anton Gauert, Michael Götz, Christian Schmitz-Linneweber

**Affiliations:** 1 Molecular Genetics, Humboldt-University Berlin, Philippstr. 13, 10115 Berlin, Germany; 2 BioEnergy GmbH, Dietersberg 1, 92334 Berching, Germany

**Keywords:** chloroplast, transgene expression, RNA processing, RNA binding, PPR, pentatricopeptide repeat, RNA stability, organelle, *Nicotiana tabacum*

## Abstract

Human papillomavirus (HPV) infection is the leading cause of cervical cancer, and vaccination with HPV L1 capsid proteins has been successful in controlling it. However, vaccination coverage is not universal, particularly in developing countries, where 80% of all cervical cancer cases occur. Cost-effective vaccination could be achieved by expressing the L1 protein in plants. Various efforts have been made to produce the L1 protein in plants, including attempts to express it in chloroplasts for high-yield performance. However, manipulating chloroplast gene expression requires complex and difficult-to-control expression elements. In recent years, a family of nuclear-encoded, chloroplast-targeted RNA-binding proteins, the pentatricopeptide repeat (PPR) proteins, were described as key regulators of chloroplast gene expression. For example, PPR proteins are used by plants to stabilize and translate chloroplast mRNAs. The objective is to demonstrate that a PPR target site can be used to drive HPV L1 expression in chloroplasts. To test our hypothesis, we used biolistic chloroplast transformation to establish tobacco lines that express two variants of the HPV L1 protein under the control of the target site of the PPR protein CHLORORESPIRATORY REDUCTION2 (CRR2). The transgenes were inserted into a dicistronic operon driven by the plastid rRNA promoter. To determine the effectiveness of the PPR target site for the expression of the HPV L1 protein in the chloroplasts, we analyzed the accumulation of the transgenic mRNA and its processing, as well as the accumulation of the L1 protein in the transgenic lines. We established homoplastomic lines carrying either the HPV18 L1 protein or an HPV16B Enterotoxin::L1 fusion protein. The latter line showed severe growth retardation and pigment loss, suggesting that the fusion protein is toxic to the chloroplasts. Despite the presence of dicistronic mRNAs, we observed very little accumulation of monocistronic transgenic mRNA and no significant increase in CRR2-associated small RNAs. Although both lines expressed the L1 protein, quantification using an external standard suggested that the amounts were low. Our results suggest that PPR binding sites can be used to drive vaccine expression in plant chloroplasts; however, the factors that modulate the effectiveness of target gene expression remain unclear. The identification of dozens of PPR binding sites through small RNA sequencing expands the set of expression elements available for high-value protein production in chloroplasts.

## 1. Introduction

Molecular pharming, or the production of recombinant proteins in plants for pharmaceutical use, has several benefits over traditional bioreactor systems [1]. For example, the scalability of production, the wide availability of agricultural skills, and the possibility of oral delivery of formulated products in edible plants make molecular pharming an attractive option for developing countries. Tobacco is a commonly used model plant for expressing transgenes due to its ease of transformation and high yields [2]. Additionally, being a non-food, non-feed crop, it reduces the regulatory procedures required to a minimum as there is a limited risk of foreign proteins entering the food chain [2]. The expression of recombinant proteins from the plastid genome in tobacco also has several advantages [3,4]. The plastid genome is a prokaryotic system, which allows for the stacking of multiple transgenes in artificial operons and simultaneous expression [5,6]. Additionally, plastid-expressed proteins are not subject to gene silencing or other epigenetic mechanisms that could disturb stable transformation [7], and high protein yields can be achieved due to plastid polyploidy [8,9]. Furthermore, the stable transformation of plastids is straightforward and precise via homologous recombination [10], and plastid inheritance is maternal, which reduces the scope of regulatory procedures [11,12].

Chloroplast gene expression is largely controlled post-transcriptionally [13]. Key factors for transcript maturation, stability, and translation are pentatricopeptide repeat (PPR) proteins [14]. The PPR protein family, with over 400 members in angiosperms, is named after a 35 amino acid motif that is repeated in tandem up to 30 times [14]. These stacked PPR repeats form an extended RNA-interaction surface that enables PPR proteins to bind their targets with high specificity and affinity [14]. The affinity for its target sequence is so high that the protection of the bound nucleotides leads to the accumulation of short RNA fragments (sRNAs) that accumulate to high amounts in chloroplasts [15]. The biological functions of RNA binding by PPR proteins are manifold, including splicing, RNA editing, RNA stabilization, and translation [16]. PPR proteins bound within 5′-untranslated regions (UTRs) often support translation of the downstream open reading frame (ORF), making them interesting factors for transgene expression in chloroplasts. One commonly used expression element in chloroplast transgenics is the intercistronic expression element (IEE) [5,6]. Originally thought to aid intercistronic cleavage of polycistronic transcripts via RNA structure, the IEE turned out to be a binding site for a PPR-like protein named HIGH CHLOROPHYLL FLUORESCENCE 107 (HCF107) [17]. Using the IEE leads to relatively high expression of transgenes, but can be problematic if used multiple times in a stacked transgene approach driven by a strong promoter. Such an over-expression of the HCF107 binding site leads to the depletion of HCF107, which in turn leads to impairment of the expression of its endogenous mRNA target [17]. Therefore, for the development of an alternative IEE, other PPR protein target sites have recently been suggested for use as efficient expression elements, especially the PPR protein CHLORORESPIRATORY REDUCTION2 (CRR2) [17]. CRR2 stabilizes the chloroplast *rps7* and *ndhB* transcripts [18]. Null mutants of CRR2 fail to accumulate mRNAs with 5′ and 3′ ends in the *rps7*-*ndhB* intergenic spacer, which results in loss of the NDH complex [18]. CRR2 is not essential for photosynthesis under normal conditions [18], and the disruption of the CRR2 target mRNA, *ndhB*, does not show any macroscopic phenotype either [19]. Like other PPR proteins, CRR2 causes the accumulation of a small RNA that corresponds to its binding site in the 5′-UTR region of *ndhB* [20], but also causes sRNA accumulation for other transcripts, albeit to a lesser extent [20]. Notably, *ndhB* sRNA is one of the most abundant chloroplast sRNAs [21], which suggests that CRR2 is a more abundant PPR protein. This could circumvent the aforementioned depletion problem that occurred when using the HCF107 binding site as an IEE.

Infection with HPV is the most common viral infection of the reproductive tract in humans and is responsible for over 610,000 new cancer cases worldwide each year [22,23], representing 5% of all new cancer cases. However, there is substantial variation in this ratio in specific geographic regions and levels of development. For example, 14.2% of all new cancer cases in sub-Saharan Africa and 15.5% of all cases in India are related to HPV infection, compared to 1.6% in North America. Cervical cancer, caused by HPV infection in 100% of cases, is the third most common type of cancer in women [24]. To date, over 200 different types of HPV have been identified, and they are classified according to their carcinogenic potential into low- and high-risk types [25]. HPV types 16B and 18 are classified as high-risk types and are the most prevalent in women infected with HPV [26,27]. HPV second-generation vaccines use virus-like particles (VLPs) to induce an immune reaction and establish immune memory for the targeted infection [28]. VLPs contain no infectious genomic material but mimic the morphology of a virus. Many viral proteins self-assemble spontaneously into VLPs [29]. For HPV vaccines, a major structural protein of the capsid, the protein L1, is used [30]. This protein has the highest conservation of all papillomavirus proteins. When expressed, it self-assembles into a VLP that mimics the native spherical virion of ~50–55 nm in diameter and is known to be immunogenic. Constitutive expression of L1 has been achieved before using artificial expression elements [30,31,32].

We here demonstrate that the CRR2 sRNA sequence can be used to express a high-value transgene, a vaccine candidate against the human papillomavirus (HPV).

## 2. Experimental Design

### 2.1. Plant Growth

*Nicotiana tabacum* transgenic lines were grown on soil with a 16 h light/8 h dark cycle at 27 °C in a CLF growth cabinet at 350 µmol·m^−2^·s^−1^. Transgenic control lines (RV5) with the *aminoglycoside-adenyltransferase* (*aadA)* cassette inserted in a neutral site without additional L1 sequences were described previously [33].

### 2.2. Vector Construction

A tobacco plastid DNA fragment encompassing the intergenic region between the *psbE* operon and *petA* of 1814 bp, that is, nucleotide positions 65,310–68,985 (GenBankTM/EBI data bank accession number Z00044.2) was amplified using primers bbfor and bbrev (Supplementary Table S1). The PCR product was restriction digested with *Sal*I and cloned into the *Sal*I site of the pBSK+ vector (Stratagene/Merck, Darmstadt, Germany). A chimeric *aadA* gene [34] was inserted into the *EcoR*V site within the intergenic region between *petA* and the *psbE* operon at position 66,058. The *aadA* cassette is in the same transcriptional orientation as the *psbE* gene. This vector was then linearized with *Hind*III, which separates the *aadA* coding sequence from the downstream *Chlamydomonas rbcL* 3′ regulatory sequence. LTB::HPV16B and HPV18 sequences were synthesized together with the sRNA sequence of *ndhB* gene; nucleotide positions 67,007–67,030; accession number Z00044.2 (GeneArt, Regensburg, Germany), respectively. The fragments containing *ndhB* sRNA and LTB:HPV16B or HPV18 were excised from their vectors by using *Hind*III restriction enzyme. This resulted in plasmids p*ndhB*::LTBL1HPV16B, p*ndhB:*:L1HPV18. The plasmids were sequenced and used for chloroplast transformation.

### 2.3. Chloroplast Transformation

Tobacco plastids were transformed by particle bombardment as described previously [10]. Specifically, we bombarded leaves of 14-day-old axenically-grown *N. tabacum* Petit Havana seedlings with plasmid DNA-coated gold particles using a gene gun (PDS-1000/He system; Bio-Rad, Feldkirchen, Germany). Spectinomycin-resistant shoots were selected on RMOP medium containing 500 mg/L spectinomycin dihydrochloride. Integration into the plastid genome was verified by Southern blot analysis using a DNA probe generated with the primers mR21 and mR35 targeting the *trnW-petG* region adjacent to the transgene insertion site.

### 2.4. Immunoblot Analysis

Total protein was extracted from fully developed leaves of four-week-old plants and was used as previously reported [35]. A total of 5 µg of insoluble proteins and of total soluble protein, individually, (determined by a Bradford assay) were loaded into each lane. For detection of the L1 protein, the MD2H11 antisera was used (DKFZ Heidelberg, Heidelberg, Germany). For quantification purposes, recombinant HPV-L1 protein from Abcam was used, ab119881. Baculovirus-derived purified VLPs (DKFZ Heidelberg) were used as a positive control.

### 2.5. RNA Extraction and RNA Gel Blot Analysis

Total RNA was extracted from leaves of four-week-old plants, powdered in liquid nitrogen using Trizol (Thermo Fisher, Hennigsdorf, Germany) according to the manufacturer’s protocol. Total RNA (4 µg) was fractionated on 1.2% agarose gels containing 1.2% formaldehyde, blotted and hybridized with radiolabeled RNA probes produced by T7 in vitro transcription from PCR products generated with primer combinations described in Table S1.

## 3. Results

### 3.1. Plant Lines Expressing Enterotoxin-L1 Fusion Proteins Show Impaired Chloroplast and Leaf Development

**Figure 1:**
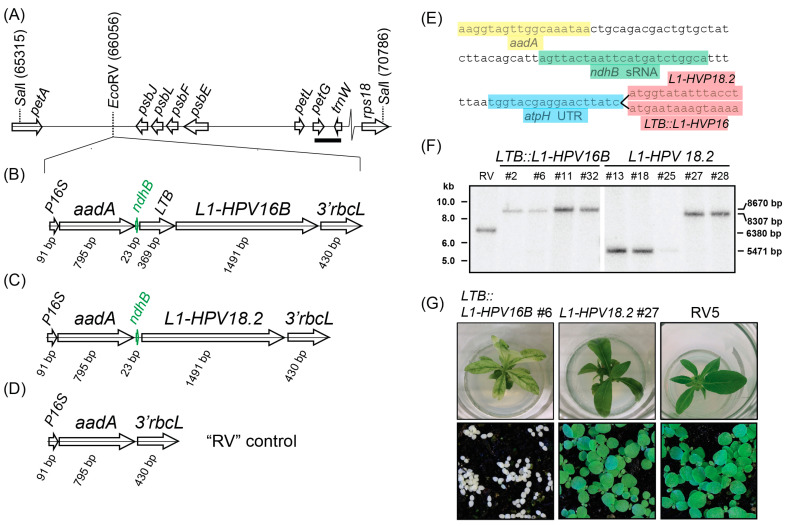
Southern blot analysis of transplastomic lines. (**A**) Schematic representation of the insertion locus. Numbers refer to positions on the tobacco chloroplast genome, accession Z00044.2 (drawn to scale). (**B**) Schematic representation of the *LTB::L1-HPV16B* transgene cassette inserted into the neutral *EcoR*V site shown in (**A**). The *ndhB* sRNA is shown in green. P16S = promoter of the chloroplast 16S rRNA gene. *3*′*rbcL* = the 3′-UTR of the *rbcL* mRNA from *Chlamydomonas reinhardtii*. Arrows point in the direction of transcription of the respective genes. (**C**) same as in (**B**), but for the *L1-HPV18.2* transgene cassette. (**D**) same as in (**B**), but for the RV5 control construct. (**E**) Sequence excerpts from *L1-HPV18.2* and *LTB::L1-HPV16B* transgene cassettes. (**F**) RFLP analysis of transplastomic lines, where 5.0 µg of cellular DNA was fragmented by *Sal*I. Hybridization was performed with a radiolabeled probe derived from the *petG* and *trnW* genes (bar in (**A**), probe position 68,595–68,872 of Z00044.2). Numbers on the right side of the blots indicate the expected size for restriction fragments: 8670 bp = *LTB::L1-HPV16B* fragment; 8307 bp = *L1-HPV18.2* fragment; 6380 bp = RV5 fragment; 5471 bp = wt fragment. (**G**) Plants were grown on sucrose-containing MS medium for three weeks (upper row) or germinated on soil and grown for two weeks (lower row).

For plastid transformation, a modified, codon-optimized L1 protein from HPV strain *18.2* and a translational fusion of the heat-labile enterotoxin B (*LTB*) gene from *E. coli* with the L1 protein from HPV strain *16B* were cloned into a plastid transformation vector (Figure 1A–C). The *LTB::L1-HPV16B* gene fragments were placed downstream of the *aminoglycoside-adenyltransferase* (*aadA*) selective marker (Figure 1B,C). A vector without any L1 protein served as a negative control. All constructs were driven by a 16S rRNA promoter for the plastid RNA polymerase and terminated with the 3′-UTR of the *Chlamydomonas reinhardtii rbcL* gene as a transcript-stabilizing 3′-sequence element. In between the *aadA* and *L1* reading frames, the sequence of the CRR2 sRNA located upstream of the plastid *ndhB* open reading frame was placed (Figure 1B,C,E). We added 30 bp of random sequences upstream of the sRNA sequence to provide space towards the 5′-located *aadA* gene (Figure 1E). Downstream of the sRNA, we added the UTR sequence of the tobacco *atpH* gene, which can support transgene translation if additional sRNA sequences are provided upstream. Finally, the resulting cassettes were flanked by sequences that allow homologous recombination into the *petA-psbJ* intergenic spacer, which has been used before to drive transgene expression and is considered a neutral integration site within the plastid genome. These two constructs were used for biolistic transformation of leaves to introduce the transgenes into the plastid genome. Selection was carried out on spectinomycin-containing medium by cutting the bombarded leaves into 5 × 5 mm squares. More than 42 and 34 green microcalli were isolated for *LTB::L1-HPV16B* and *L1-HPV18.2*, respectively. They were propagated on fresh medium and transferred to shoot-inducing MS medium. After initial verification that the shoots contained the *aadA* cassette, selected transplastomic lines underwent three cycles of regeneration to allow for the segregation of the transgene and wild-type (wt) plastid genomes. The resulting plants were subjected to Southern analysis to determine whether homoplastomy was reached. The probe was designed against the *petG-trnW* region (bold bar in Figure 1A) and detected a *Sal*I restriction length polymorphism between the wt and the transplastomic lines. This analysis demonstrated that the *LTB::L1-HPV16B* transplastomic lines #2, #6, #11, and #32, and the *L1-HPV18* lines #27 and #28 only had the band size expected after transgene insertion, 8.7 and 8.3 kB kb, respectively, but there was no signal for smaller fragments indicative of wt plastomes. In conclusion, these lines were homoplastomic for the transgene insertion and were used for further assays. By contrast, lines *L1-HPV18.2* #13, #15, and #25 showed only a fragment corresponding to the size expected for wt fragments, which means they did not carry any transgene sequence. The “RV” control line displayed a single band of intermediate size, as expected for a homoplastomic line with an integration of the *aadA* cassette into the neutral *EcoR*V site.

Morphologically, when grown on MS medium with sugar, *LTB::L1-HPV16B* plants showed a severe loss of chlorophyll, which was mostly restricted to leaf vein areas, while the remainder of the leaves was almost white (Figure 1G and Figure S1). The plants were stunted. On soil under standard growth conditions, the plants germinated but never accumulated enough chlorophyll to be visible and did not develop primary leaves (Figure 1G). However, when grown at low light conditions, the plants were green (Figure S1), set flowers, and produced seeds. In contrast to *LTB::L1-HPV* plants, *L1-HPV18.2* lines had the same color as the wildtype plants, but sometimes had slightly narrower leaves. They were fully autotrophic and grew like wt plants on soil (Figure 1G).

### 3.2. The L1 Proteins Accumulated in the Transplastomic Lines

**Figure 2:**
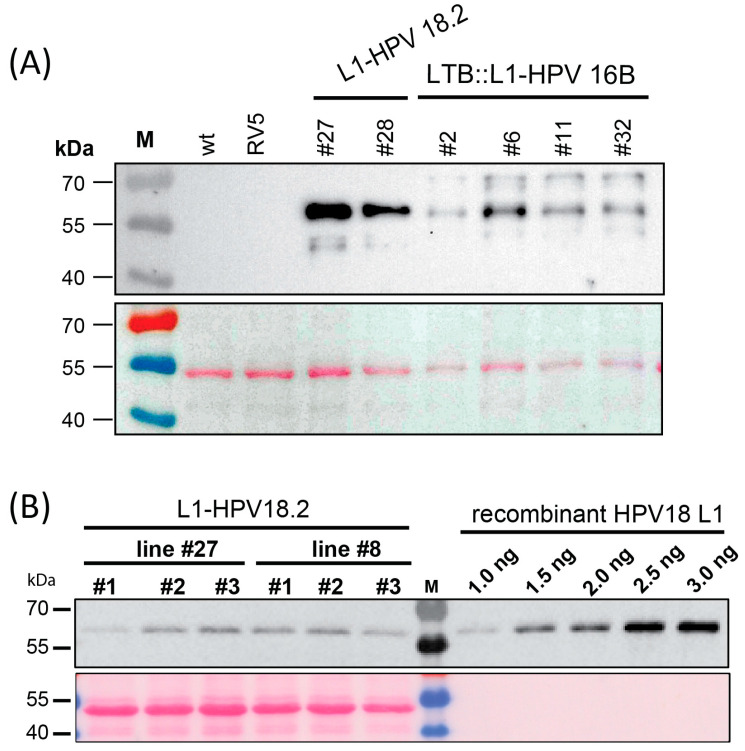
Immunoblot analysis of L1 proteins in transplastomic lines. (**A**) Extracts totaling 4 µg from transplastomic *L1-HPV18.2* and *LTB::L1-HPV16B* plants were analyzed by immunoblotting using the MD2H11 monoclonal antibody. Proteins from RV lines and wt served as negative controls. (**B**) Quantification of L1-HPV18.2 protein accumulation in transplastomic lines. Protein extraction was performed from total plant leaf tissue from plants belonging to two independent *L1-HPV18.2* transplastomic lines grown for four weeks on 0.5 MS medium with sucrose. For both lines #27 and #28, five µg of total plant protein extracts was analyzed by SDS-PAGE and blotted onto a nylon membrane. The MD2H11 monoclonal antibody directed against the L1 protein was used to probe the blot. For quantification purposes, different amounts of purified recombinant HPV18 L1 protein were loaded onto the same gel.

We tested the accumulation of the L1 and LTB::L1 proteins in the transplastomic lines using immunoblot analysis with an antibody that could detect both the HPV16B and the HPV18.2 L1 proteins. We inserted the *ndhB* sRNA upstream of the two L1 protein versions, which allowed for the production of protein from the downstream cistron. Indeed, all homoplastomic lines accumulated L1 protein in leaves (Figure 2A). This was true for green leaves from *L1-HPV18.2* lines #22 and #27, as well as for pale leaves from *LTB::L1-HPV16B* lines #2, #6, #11, and #32. The sizes of the observed signals matched the expected size of 56.5 kDa for the *L1-HPV18.2* protein (Figure 2A). For the *LTB::L1-HPV16B* protein, the expected size was 69.5 kDa. Signals of about 70 kDa were observed, but there were also additional proteins of smaller size detected (Figure 1A). This could indicate degradation of the LTB::L1-HPV16B protein. No protein was detected in samples extracted from RV5 control plants or wt plants, demonstrating the specificity of the antibody. Next, we quantified the *L1-HPV18.2* protein by comparing the soluble proteins from transplastomic plants with defined amounts of recombinant L1 protein on the same gel as reference (Figure 2B). The yield was low, with 0.02% and 0.03% total soluble protein accumulating in lines #27 and #28, respectively. Nevertheless, the immunological analysis of the transplastomic lines indicated that the L1 proteins can be expressed using the *ndhB* sRNA sequence as a post-transcriptional expression element.

### 3.3. Mono- and Dicistronic L1 mRNAs Accumulated in Transplastomic Plants

**Figure 3:**
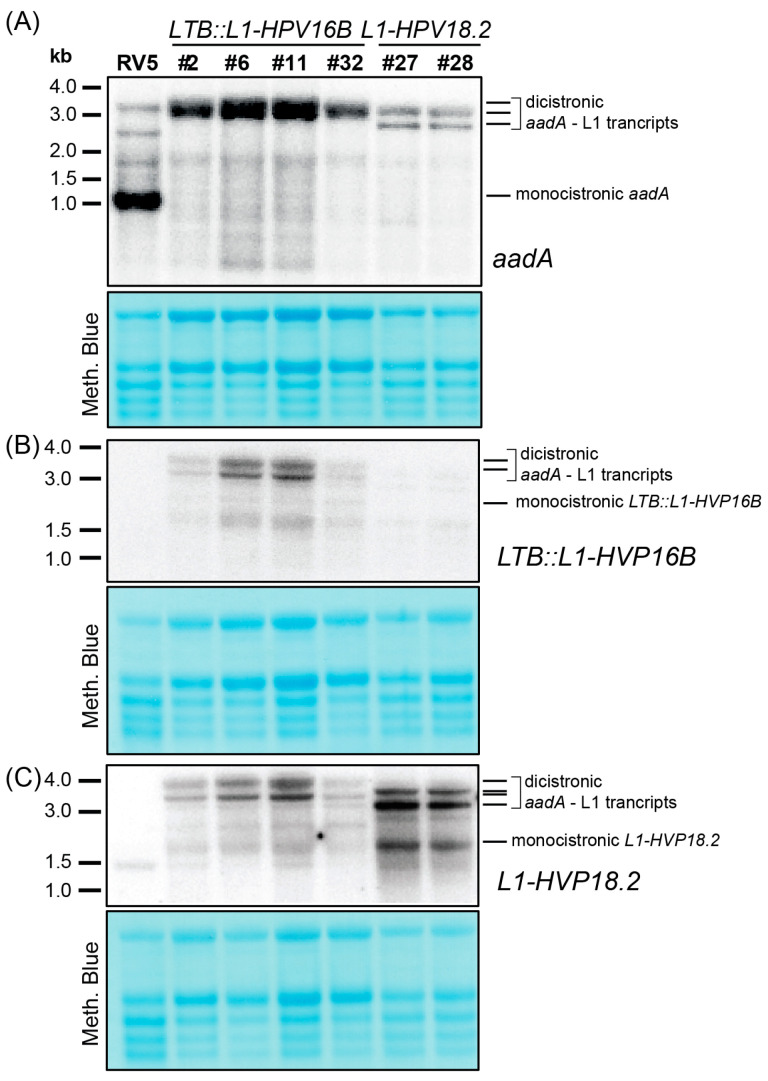
Analysis of transgenic transcript accumulation in *L1-HPV18.2* and *LTB::L1-HPV16B* lines. Four micrograms of total leaf RNA from the indicated genotypes was fractionated on 1.2% agarose gels and analyzed by hybridization to probes for the *aadA* transgene (**A**), the *LTB::L1-HPV16B* transgene (**B**), and the *L1-HPV18.2* transgene (**C**) (see Supplemental Table S1 online for probe primer sequences). Loading was controlled by staining rRNAs on the blots with methylene blue.

To examine the mRNA level of transgenes and analyze the effect of sRNA sequence on mRNA accumulation, we conducted RNA gel blot experiments using probes for the *aadA* gene and the two L1 gene isoforms. Analysis of the *aadA* mRNA revealed a strong signal corresponding to the monocistronic transcript in the RV5 control line, as well as additional higher-molecular weight bands (Figure 3A). No monocistronic *aadA* was present in any of the transplastomic lines, suggesting that the *ndhB* sRNA cannot serve as a 3′-stabilizing element for this upstream cistron. The most prominent bands observed in L1 lines were between 2.8 and 3.5 kb in size (Figure 3A–C). Based on their length and detection with both the *aadA* probe as well as the *L1* probes, we concluded that they are dicistronic and encompassed the *aadA* gene and the *L1* gene. For *LTB::L1-HPV16B* probing, the larger band likely represented a read-through transcript into the adjacent *psbE* operon (Figure 1A), while the smaller transcript corresponded to a transcript terminating at the 3′*rbcL* stabilizing sequence (Figure 3B). A similar situation was observed in the *L1-HPV18.2* lines, where a double band was also detected, but shifted down by about 0.3 kb relative to *LTB::L1-HPV16B* lines (Figure 3C), consistent with the shorter transgene length in *L1-HPV18.2* lines. In addition to these long precursor transcripts, there were also signals representing much shorter *L1*-specific transcripts in the transgenic lines. These could represent monocistronic *L1* transcripts (Figure 3B,C), but compared to the dicistronic transcripts, they were weaker, possibly indicating inefficient processing or instability.

While we designed probes specifically for *L1-HPV16B* and *L1-HPV18.2*, both probes also picked up signals in the non-targeted samples. This cross-hybridization was likely due to the related *L1* sequences present in the sample. The cross-hybridization signals were weak in the *L1-HPV18.2* lanes when probing for *LTB::L1-HPV16B* and were stronger in the *LTB::L1-HPV16B* samples when probing for *L1-HPV18.2*. The high similarity of the two probes, targeting the 5′-end of the *L1* sequence and sharing 68% nucleotide identity over a total length of 334 nt could explain the observed cross-hybridizations.

### 3.4. Increased Expression of ndhB sRNA in Transplastomic Lines

**Figure 4:**
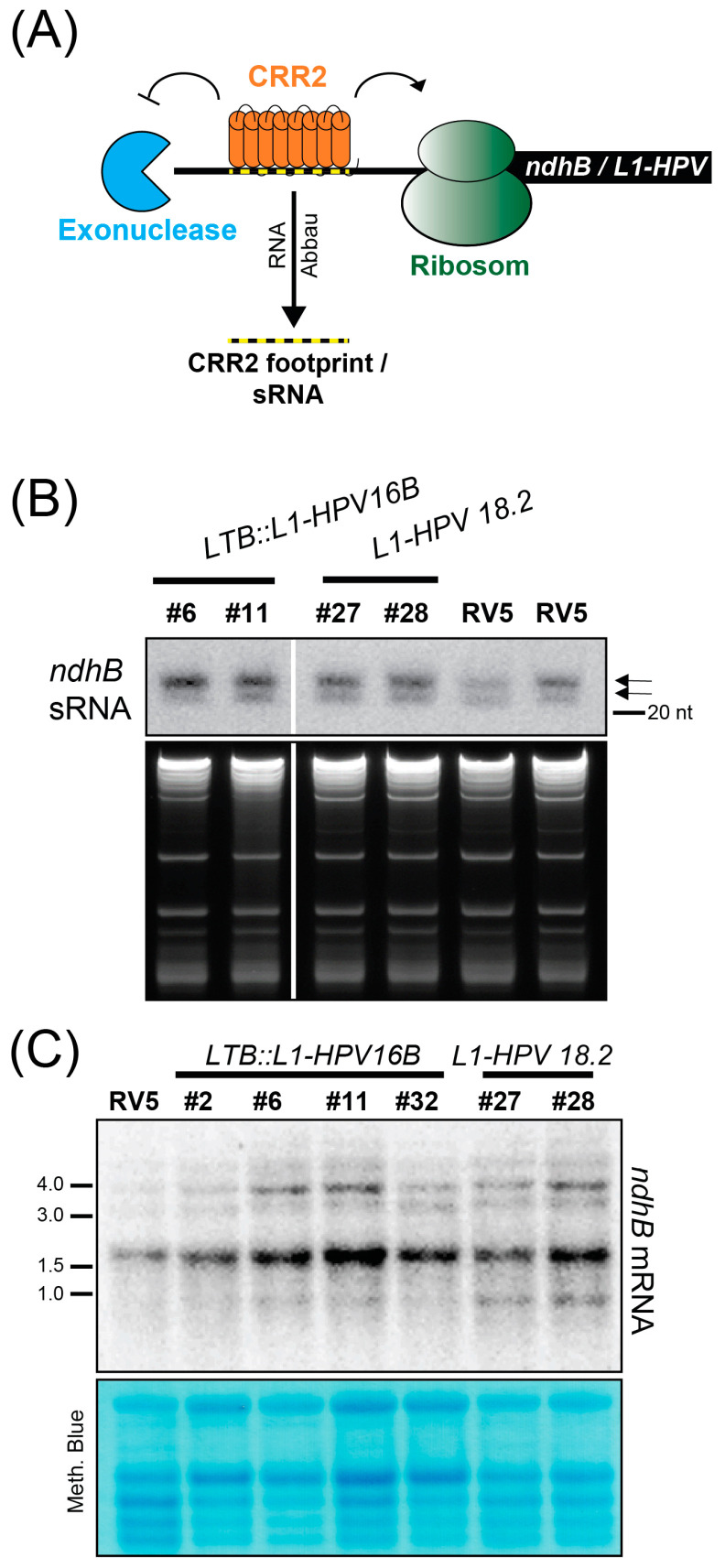
Analysis of endogenous *ndhB* sRNA and mRNA accumulation in *L1-HPV18.2* and *LTB::L1-HPV16B* lines. (**A**) Schematic representation of the proposed function of CRR2 for transgene expression. (**B**) Three µg of total leaf RNA from the indicated genotypes was separated on a denaturing PAA gel, blotted, and hybridized with end-labeled oligonucleotides complementary to the *ndhB* sRNA (oligo sequence in Supplementary Table S1). Arrows indicate the two known *ndhB* sRNA isoforms. The ethidium-bromide-stained gels are shown below to illustrate sample loading. (**C**) Four µg of total leaf RNA from the indicated genotypes was separated on a 1.2% denaturing agarose gel and transferred to nylon membrane. This is the same blot as the one used for the *L1-HPV18.2* probing in Figure 3C. After stripping of the *L1* probe, *ndhB* mRNAs were detected using a radiolabeled DNA probe generated by PCR (see Supplementary Table S1 for primers).

The inclusion of the *ndhB* sRNA sequence into the transgene cassettes in *LTB::L1HPV16B* and *L1:HPV18* was intended to attract the CRR2 protein, redirecting its ability to support the expression of the endogenous *ndhB* mRNA towards the transgenes. To measure the CRR2 activity, we decided to detect the corresponding *ndhB* sRNA, reasoning that an increased amount of target mRNA in transgenic lines could lead to an increased accumulation of the corresponding sRNA (Figure 4A). While we cannot distinguish between the footprint stemming from the endogenous *ndhB* mRNA target of CRR2 or from the transgene sequence, we can detect the amount of sRNA present in transgene lines compared to RV5 control plants. To analyze the accumulation of *ndhB* sRNAs, we conducted RNA gel blot experiments using polyacrylamide gels for better resolution in the small RNA range. In comparison to the control RV5 line, there was a stronger signal in all transgenic lines (Figure 4B). This indicates an increased number of CRR2:RNA complexes, likely due to the surplus supply of CRR2 target sites in transgene mRNAs.

To monitor the effects of *L1* transgene expression on endogenous *ndhB* mRNA levels and processing status, we analyzed *ndhB* accumulation by RNA gel blot hybridization (Figure 4C). We found several larger precursors in the range of 3–5 kb, representing co-transcripts with the genes *rps7* and *rps12* that are located adjacent to *ndhB* on the plastid chromosome. The signals at 1.6 kb corresponded to the spliced monocistronic *ndhB* mRNA (the *ndhB* reading frame is 1532 nt long). Importantly, transcript patterns were very similar between all transgenic lines and the RV5 control and there is no loss of signal in any transgenic line. This suggests that CRR2 is not titrated away from performing its endogenous function on the *ndhB* mRNA.

## 4. Discussion

We report here a simplified, dicistronic construct for the expression of the L1 vaccine against cervical cancer. Expression elements used to stabilize and translate mRNAs from transgenes in chloroplasts are often artificial sequences such as the G10L sequence of the lambda phage T7 [36]. Control of the use of such sequences is limited and often leads to constitutive transgene expression. As an alternative, PPR proteins and their target sequences have been shown in recent years to be able to drive transgene expression, offering the advantage of transgene induction by manipulating the nuclear-encoded PPR gene [37]. Both endogenous PPR proteins as well as artificial PPR proteins have been used to drive expression of marker genes such as GFP, including induced expression systems [17,37,38]. Here we applied the target sequence of the PPR protein CRR2 to the expression of the L1 protein of HPV. The L1 protein was chosen as it has been the target of several attempts at expression in plants already, making comparisons with more standard expression vectors straightforward [39]. Additionally, the L1 protein is highly effective as a vaccine against HPV, a continuous public health problem as a sexually transmitted disease and a critical factor in the pathogenesis of various cancers [40]. HPV infection rates are much higher in developing countries than in developed regions [23,41]. Production of L1 in plants could make vaccines more easily accessible in developing countries, where fermenter-based approaches are more difficult to implement. Delivery of a vaccine orally via edible, L1-producing plants would be a practical solution for limited technical infrastructure for standard production of a recombinant protein for intravenous vaccination in developing regions.

Our study shows that it is possible to express the L1 protein using a construct that includes the target sRNA sequence of the CRR2 protein. We did not observe titration effects on the endogenous *ndhB* mRNA similar to that previously suggested for the overexpression of the target sequence of the HCF107 RNA-binding protein on the *psbH* mRNA [17]; *ndhB* mRNA and sRNA patterns in transplastomic lines were in line with published transcript patterns for tobacco *ndhB* [20,42]. However, the protein yield was low compared to previous attempts using standard expression vectors, where 1.5% L1 or more of total soluble protein was achieved [31,32,43,44], while the yield in our study is much lower. In an earlier experiment, the CRR2 footprint was able to drive the expression of GFP effectively despite weak promoter activity [17]. This suggested that CRR2 is able to protect RNAs from exonucleolytic degradation both from the 5′ and 3′ ends and also to support translation of the downstream cistron [17]. The low yield of L1 protein in our study may be due to differences in the sequence context of the CRR2 sRNA. The immediate downstream sequence of the CRR2 footprint was the same as in a previous study, but the more distal sequences were distinct, with a different preceding cistron (*neomycin* instead of *aadA*) and the *GFP* cistron following downstream instead of *L1* [17]. We, therefore, speculate that differences in long-range RNA structure formation could impact the availability of the CRR2 target sequence and thus prevent its action. This would be in line with the rather low amount of monocistronic *L1* mRNA despite strong accumulation of the dicistronic *aadA-L1* transcript (the latter including long transcripts stemming from read-through transcription activity [45]) as well as the only mild increase in *ndhB* sRNA in transplastomic lines. An effective association of CRR2 with the dicistronic *aadA-L1* mRNAs would be expected to produce copious amounts of both RNA species. An alternative explanation for the low yield is that the protein stability of L1-HPV18.2 and LT:L1-HPV16.2 is lower than that of the GFP marker. However, given that L1 proteins have been successfully expressed in various sequence contexts and fusions and accumulate to high levels [39], this explanation is less likely than a protein production problem.

The data presented here show another unexpected result when compared to the previous usage of the CRR2 sRNA sequence. We found that the CRR2 sRNA is not able to stabilize the upstream monocistronic *aadA*. In previous research, monocistronic upstream *neo* RNA was easily detected in RNA gel blot hybridizations, which led to the conclusion that CRR2 could block 3′-to-5′ exonucleolytic degradation of RNA [17]. However, this is not the case in the current study. The differences in sequence between the expression constructs are likely the reason for this. To conclude, while the CRR2 sRNA-containing construct can support L1 production, the sequence context, such as the markers and transgenes used, significantly impacts yield.

In order to increase the immunogenicity of the L1 protein, we attempted to express a translational fusion of L1 with the *E. coli* enterotoxin LTB in chloroplasts, following previous research [32,43,46]. The *HPV16B* sequence used in this study lacks the first 10 N-terminal amino acids and two cysteine residues were replaced by serines. This modification prevents the assembly of virus-like particles and retains L1 capsomers, which are more thermostable than higher-order assemblies [32,43,47]. Despite accumulating less protein than L1:HPV18.2, the effect on the phenotype was drastic. The plants were almost completely pale and strongly inhibited in growth on soil, while they grew slowly on MS-medium with sugar—they had lost their photosynthetic ability. In contrast, the *L1-HPV18.2* lines had a normal phenotype. Additional plants that grew on spectinomycin-containing medium, but did not contain the transgene nor the *aadA* marker were considered mutants in the plastid rRNA or other ribosomal components that are known to give rise to spectinomycin-resistance [48,49]. The cause of the toxic effect in *LTB::L1-HPV16B* plants is currently unclear. LTB enterotoxin has been expressed in tobacco chloroplasts successfully before, at levels more than 2% TSP and thus much higher protein levels than in our *LTB::L1-HPV16B* line without detrimental phenotypes reported [50,51,52]. Likewise, L1-HPV16B including the mutated cysteines has been expressed successfully in tobacco chloroplasts, again with no detrimental phenotype reported (e.g., [32,53]). It is possible that the fusion of LTB and L1-HPV16B is behind the observed plant developmental effects, despite the fact that fusions of L1 with other epitopes are non-toxic [53]. Additionally, fusions of the cholera toxin B, which differs by only four amino acids from LTB, with various proteins, did not negatively affect chloroplasts (reviewed in [54]). An identical fusion of LTB with L1 of HPV16B did lead to chlorotic leaves, growth retardation, and male sterility [32], but did not show the full bleaching of seedlings we observed here. Overall, only a few differences from previous attempts at expressing the L1-HPV protein led to dramatic phenotypic alterations. This demonstrates that we still do not fully understand the rules for designing transgenes in complex, polycistronic contexts within the chloroplasts. Recent advances in generating inducible systems for chloroplast transgenes [55], including inducible PPR proteins [37] will likely help to avoid toxic effects.

## 5. Conclusions

This study has provided evidence that sRNA sequences of PPR proteins can be used for the expression of an HPV vaccine. However, there are differences in protein production efficiency and target RNA processing in different constructs that utilize the CRR2 sRNA that are not yet understood. The next step will be to investigate the impact of sequence context, possibly through the formation of differential RNA structure on expression efficiency. If this can be resolved, the many known PPR protein-related sRNAs can be a powerful toolset for adjusting transgene expression in chloroplasts.

## Data Availability

Data are contained within the article or Supplementary Material.

## Supplementary Materials

The following supporting information can be downloaded at https://www.mdpi.com/article/10.3390/metabo13030315/s1, Table S1: Oligonucleotides used in this study. Figure S1: Phenotype of *LTB::L1-HPV16B* plant lines grown under low light conditions.
