# Supplementary material for: A CRR2-Dependent sRNA Sequence Supports Papillomavirus Vaccine Expression in Tobacco Chloroplasts
Source: Metabolites. 2023 Feb 21;13(3):315. doi: 10.3390/metabo13030315 (PMC10054877; doi:10.3390/metabo13030315)
Supplement: Supplementary file 1 [file metabolites-13-00315-s001.zip › supplementary material/metabolites-2092288-supplementary figure s1.pdf]

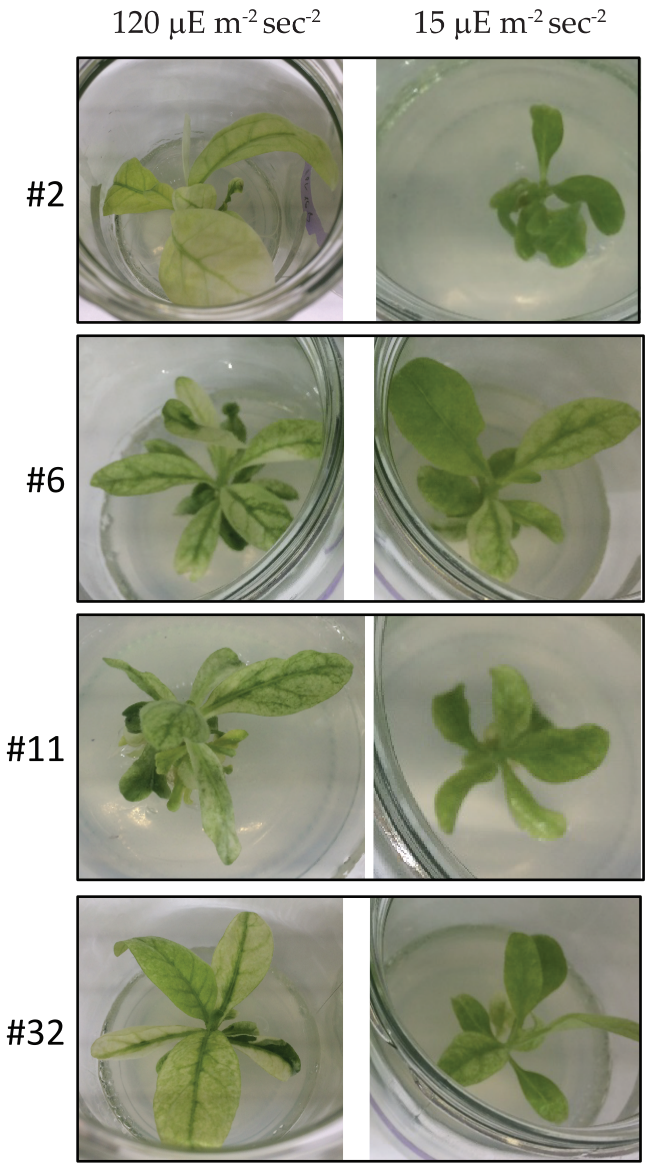

Figure S1: Phenotype of *LTB::L1-HPV16B* plant lines grown under low light conditions.

Plants were grown on sucrose-cotaining MS medium for three weeks either at 120 μE m^{-2} sec^{-2} or at 15 μE m^{-2} sec^{-2}.
