# Supplementary material for: A CRR2-Dependent sRNA Sequence Supports Papillomavirus Vaccine Expression in Tobacco Chloroplasts
Source: Metabolites. 2023 Feb 21;13(3):315. doi: 10.3390/metabo13030315 (PMC10054877; doi:10.3390/metabo13030315)
Supplement: Supplementary file 1 [file metabolites-13-00315-s001.zip › supplementary material/metabolites-2092288-supplementary table s1.pdf]

Table S1. Oligonucleotides used in this study.

Name	Sequence	Application
AndhB	GTCGTTGCTTTTCTTTCTG	Northern
AT7ndhB	TAATACGACTCACTATAGGGAAATATAGGCCTGCCT	Northern
aadAfw1	GGAAACTTCGGCTTCCCCTGG	Northern, sequencing
aadAT7	GTAATCGACTCACTATAGGGAACCGGATCAAAGAGT	Northern
HVP16B_forward	ATGGACCTGGACCTGGAATG	Northern, sequencing
T7 HVP16B_reverse	TAATACGACTCACTATAGGGCTCTTCCTACTTCTACTCCT	Northern
HVP182_forward	CTGCTGGAGGAGGAAATAAAC	Northern, sequencing
T7 HVP182_reverse	TAATACGACTCACTATAGGGGCAGGAGCACATCCTAAAATAC	Northern
mR21	AGAACGTGGGTCTCCAAAAC	Southern
mR35	TGGAATCGTGTTAGGTCTAATTCC	Southern
bbfor	GATTTGTCGACATCAAGTTCG	cloning
bbrev	ACAGGAGGACGTTCGTTTGC	cloning
pHVP16rev	TTATTACAAGCTGGATTAAAAGC	cloning; genotyping; sequencing
pHVP18rev	AAGCTGGATTAAGAAGAAAACC	cloning; genotyping; sequencing
aadoutrev	ACTGCGGAGCCGTACAAATG	sequencing
